# Competition between Structural Relaxation and Crystallization in the Glass Transition Range of Random Copolymers †

**DOI:** 10.3390/polym12081778

**Published:** 2020-08-08

**Authors:** Jürgen E. K. Schawe, Claus Wrana

**Affiliations:** 1 Mettler-Toledo GmbH—Analytical, Heuwinkelstrasse 3, 8606 Nänikon, Switzerland; 2 Compounds AG, Barzloostrasse 1, 8330 Pfäffikon, Switzerland; claus.wrana@compounds.ch

**Keywords:** crystallization kinetics, structural relaxation, random copolymer, self-assembly confinement, fast scanning calorimetry, rigid amorphous fraction

## Abstract

Structural relaxation in polymers occurs at temperatures in the glass transition range and below. At these temperatures, crystallization is controlled by diffusion and nucleation. A sequential occurrence of structural relaxation, nucleation, and crystallization was observed for several homopolymers during annealing in the range of the glass transition. It is known from the literature that all of these processes are strongly influenced by geometrical confinements. The focus of our work is copolymers, in which the confinements are caused by the random sequence of monomer units in the polymer chain. We characterize the influence of these confinements on structure formation and relaxation in the vicinity of the glass transition. The measurements were performed with a hydrogenated nitrile-butadiene copolymer (HNBR). The kinetics of the structural relaxation and the crystallization was measured using fast differential scanning calorimetry (FDSC). This technique was selected because of the high sensitivity, the fast cooling rates, and the high time resolution. Crystallization in HNBR causes a segregation of non-crystallizable segments in the macromolecule. This yields a reduction in mobility in the vicinity of the formed crystals and as a consequence an increased amount of so-called “rigid amorphous fraction” (RAF). The RAF can be interpreted as self-assembled confinements, which limit and control the crystallization. An analysis of the crystallization and the relaxation shows that the kinetic of both is identical. This means that the Kohlrausch exponent of relaxation and the Avrami exponent of crystallization are identical. Therefore, the crystallization is not controlled by nucleation but by diffusion and is terminated by the formation of RAF.

## 1. Introduction

The complex interactions in and between polymer chains cause a metastable semicrystalline structure, which consists of an amorphous and a crystalline fraction (CF) [1,2,3,4,5]. The molecular mobility in the remaining amorphous fraction is heterogeneously distributed. A model to express this situation is the separation of the non-crystalline component in a mobile amorphous fraction (MAF) and a so-called rigid anamorphous fraction (RAF) only. The MAF contributes to the glass transition. The molecular mobility in the RAF is reduced [6,7,8,9,10,11].

The complex interaction between structure, molecular constitution, and configuration as well as the local molecular mobility determines the crystallization kinetics [12,13,14,15,16]. The complexity of the polymer crystallization becomes evident if the crystallinity, the crystal size, and the melting temperature are discussed as a function of the crystallization temperature [17,18,19,20,21]. At a decreased crystallization temperature, the formed crystallites are less stable, which yields a reduced melting temperature. A drawback for the characterization with Differential Scanning Calorimetry (DSC) is the possibility of the reorganization of crystals during the measurement [22,23,24,25,26,27]. This effect can be reduced by an increased heating rate [20,28,29].

An important aspect of the crystallization at quiescent conditions is the crystallization with a reduced diffusion at temperatures in the vicinity of the glass transition. Structural relaxation occurs in the glassy state if the cooling from the melt is performed sufficiently fast [30,31,32,33]. During this process, precursors for crystals can be formed. This observation was reported the first time for poly(ethylene terephthalate) (PET) by Yeh and Geil [34]. Later, it was shown for various polymers that annealing in the glassy state accelerates the subsequent cold crystallization [35,36,37,38,39]. Crystal nuclei form during annealing in the glassy state. A real crystallization process during annealing below the glass transition has been detected for poly(butylene succinate) by DSC and Temperature-Modulated DSC (TMDSC) [40]. Schick and co-workers studied the behavior during annealing for various crystallizable polymers by Fast Differential Scanning Calorimetry (FDSC) [41,42,43,44,45]. These studies indicate that the structure formation during annealing in the glass transition range follows a sequential order. After the completion of the structural relaxation, the occurrence of the homogeneous nucleation is followed by the crystals’ growth [46].

Another aspect of polymer crystallization can be studied in confinements, which are formed in micro domains in phase-separating blends and in block copolymers [47,48]. At sufficiently small domain sizes, crystallization shifts to lower temperatures, the crystal size is reduced, and the nucleation and growth mechanism may differ compared to bulk crystallization [49].

Hu et al. [50] recently published a review of the crystallization behavior of random copolymers. For homogeneous random copolymers, microphase separation occurs upon crystallization due to monomer segregation (caused by the chemical difference of the monomers) and monomer sequence segregation (according to the sequence length of identical monomer units). Random copolymers consist of two different monomers A and B in which only monomer A has the tendency to crystallize. The equilibrium melting temperature, *T*_eq_, can be described by

(1) $$\frac{1}{T_{\mathrm{eq}}}=\frac{1}{T_{A}^{0}}-\frac{R}{\Delta h_{A}^{0}}\ln\;X_{A}$$

where *T*_f_^0^ and Δ*h*_A_^0^ are the equilibrium melting temperature and the molar equilibrium melting enthalpy of the homopolymer A. *X*_A_ is the mole fraction of the monomer A, and *R* is the gas constant [51]. The melting temperature of truly random copolymers depends on the content of monomer A. At low *X*_A_, the melting temperature is close to the glass transition. In such a case, the size of the formed crystals is quite small [50]. Therefore, random copolymers with a low fraction of A are ideal materials to study the structural changes during annealing around the glass transition range.

To our knowledge, the competition between structural relaxation and the crystallization behavior in random copolymers has not yet been investigated systematically. Therefore, we focus on the crystallization kinetics of the expected small crystalline structures and the formation of a RAF as the consequence of the microphase separation due to crystallization. In addition, we were interested in the validation of the sequential order of structural relaxation, nucleation, and crystallization for random copolymers with a low crystallization temperature.

In this report, fully hydrogenated nitrile-butadiene copolymer (HNBR) was selected as an example for a crystallizable random copolymer. For comparison, also the feedstock for the synthesis of HNBR, the amorphous nitrile-butadiene copolymer (NBR) was investigated. The measurements were performed using fast differential scanning calorimetry (FDSC) due to the improved time resolution and the opportunity to characterize HNBR in the amorphous state. For additional measurements, a conventional DSC was used.

## 2. Materials and Methods

### 2.1. Materials

**Figure 1:**
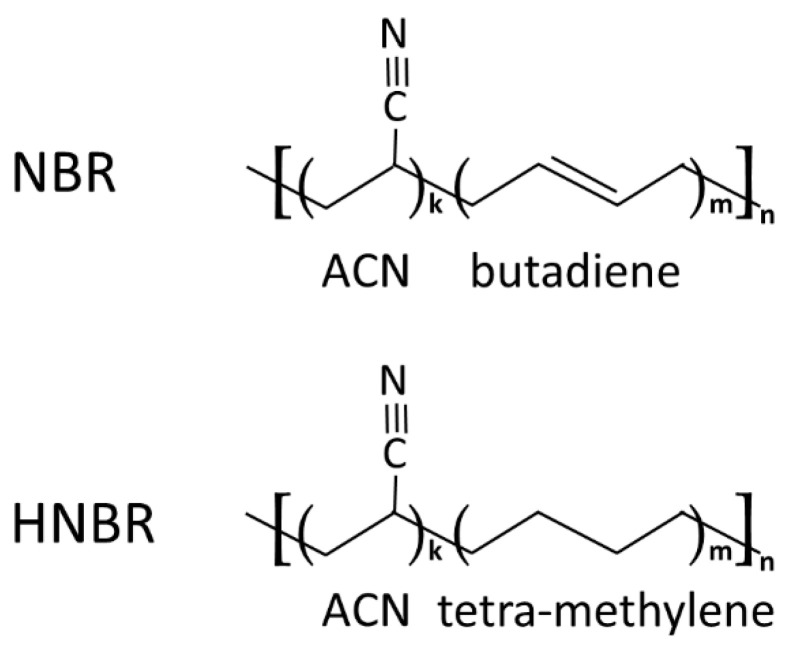
Simplified chemical structure of nitrile-butadiene copolymer (NBR) and fully hydrogenated NBR (HNBR). In each acrylonitrile (ACN)-butadiene or ACN-tetra-methylene sequence, k and m are random numbers.

A Nipol 1082 V from Zeon Corp. (Chiyoda, Japan) with an acrylonitrile (ACN) content of 34 wt % (34.4 mol%) was chosen as a typical copolymer of acrylonitrile and butadiene (NBR). The 1.4-cis butadiene isomer content is approximately 80%. The polybutadiene segments consist of typically 6–8% vinyl butadiene [52]. Therban 3407 from Lanxess A.G. (Köln, Germany) is an HNBR with a comparable macromolecular structure with 34 wt % (35.2 mol%) ACN. Both materials are random copolymers. In Figure 1, the schematic chemical structures of the polymers are shown.

### 2.2. Conventional Differntial Scanning Calorimete (DSC)

For conventional DSC measurements, a DSC 3+ (Mettler-Toledo GmbH, Nänikon, Switzerland) was used. The device was equipped with an HSS 9+ sensor and an Intra-Cooler (TC100 from Huber Gältemaschinenbau AG, Offenburg, Germany). The DSC cell was purged with nitrogen (40 mL/min). A nitrogen flow of 100 mL/min was taken as protective gas to prevent freezing. The temperature and enthalpy calibration was performed with *n*-octane, water, and indium. For each temperature, the thermal lag was determined according to the operation instruction. The samples (typical sample mass of 10 mg) were encapsulated in hermetically sealed 40 μL standard Al-crucibles.

### 2.3. Fast Differential Scanning Calorimetry (FDSC)

The FDSC technique enables cooling and heating measurements at fast rates up to several thousand Kelvin per second. To reach such high scanning rates, a removable MEMS chip sensor is used (MEMS: microelectromechanical system). The chip sensor consists of a thin silicon-nitrate/silicon-oxide membrane with two embedded DSC furnaces. The active zone (furnace) of the USF-1 sensor has a diameter of 500 μm. The sensor has a nominal temperature range up to 450 °C and can be used up to 520 °C. The sensor is described in reference [53]. The UFS-1 sensor was installed in a Flash DSC 1 (Mettler-Toledo GmbH, Nänikon, Switzerland), which is connected with a TC100 Intra-Cooler (Huber Kältemaschinenbau AG, Offenburg, Germany). The ready temperature of −95 °C was selected, and the Flash DSC was purged with a nitrogen gas flow of 30 mL/min. Details of this technique including low-temperature calibration are reported elsewhere [19,54,55].

### 2.4. Sample Preparation for FDSC

To reach the fast scanning rates in FDSC, small samples with a good thermal contact to the sensor are required. Due to the static and dynamic temperature gradients, the sample thickness should not exceed 10 μm [56,57]. The requirements of the sample preparation and an operation method for vulcanized [55] and un-vulcanized rubbery polymers [58] was discussed recently. According to these findings, small amounts of the material were placed with a single hair on the center of the active zone of the sensor and heated to 200 °C. To achieve a homogenization of the sample thickness and to generate of a good thermal contact, the sample was stored at this temperature for 3 min. Too high samples were flattened on the sensor using a thin and soft copper wire.

The determination of the sample mass for FDSC measurements is an important task for quantitative evaluations [59,60,61]. If amorphous polymers are characterized, the sample mass is estimated using the intensity of the glass transition. In a first step, the change of the specific heat capacity at the glass transition, Δ*c_p,_* is measured with a conventional DSC. In the next step, the step height of the heat flow at the glass transition, ΔΦ_Tg_, is determined by FDSC. Finally, the sample mass can be estimated by

(2) $$m=\frac{{\Delta \Phi }_{\mathrm{Tg}}}{\beta \;\Delta c_{p}}$$

where *β* is the scanning rate of the FDSC measurement. The mass of the FDSC samples was in the range between 100 and 1000 ng with an experimental uncertainty of approximately 10%.

## 3. Results and Discussion

### 3.1. NBR and HNBR by Conventional DSC

#### 3.1.1. Characterization of the Materials

**Figure 2:**
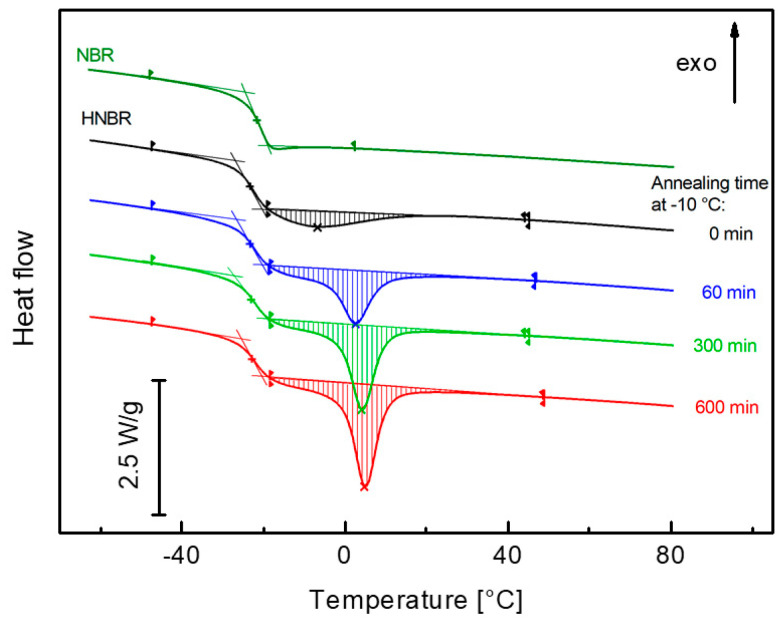
Differential Scanning Calorimetry (DSC) curves of HNBR and NBR (second run). The glass transition and melting peaks are evaluated. HNBR was annealed at −10 °C.

Conventional DSC measurements are performed with a heating rate of 10 K/min. The samples were previously cooled from 100 to −80 °C with 20 K/min. The heating curves are shown in Figure 2. For HNBR, the glass transition temperature is *T*_g_ = −24.0 °C and the intensity is Δ*c_p_* = 0.426 J/(g K). Before the heating measurement, the sample was not completely amorphous, the melting peak appears directly after the glass transition with an enthalpy of Δ*h*_m_ = 5.24 J/g (Figure 2). The crystals formed during previous cooling with 20 K/min. After annealing at −10 °C, the melting peak increases, and the peak temperature shifts to 5 °C.

The shape of the glass transition in copolymers is an indication for the nano- and microstructure. Block copolymers tend to show phase separation, and two glass transitions can be observed. As a result of structural variations, gradient copolymers show a relatively broad glass transition. In contrast, random copolymers, such as the investigated polymers, have a single narrow glass transition with the width below 20 K. This is also characteristic for homopolymers and indicates the absence on nano- and microphase segregation [62,63]. This agrees with the DSC measurements of NBR and HNBR. In addition, dielectric measurements in HNBR showed only a single non-structured main relaxation process [64]. A separate relaxation of acrylonitrile-rich domains was not found. The enthalpy peaks after annealing at −10 °C are not caused by enthalpy relaxation but by crystallization. The amount of enthalpy production is too large for a structural relaxation.

The composition of the crystals formed in HNBR depend on the ACN content. For HNBR with about 50% ACN, the crystals contain dyads of acrylonitrile and tetramethylene sequences [65,66]. At lower ACN contents, below approximately 35%, the crystals are only formed by tetra-methylene sequences [66,67]. No crystallization occurs in between. This means that the chemical structure of the crystals in the material of the investigation are comparable to polyethylene (PE) crystals and the equilibrium melting enthalpy can be assumed to be identical to PE (Δ*h*_PE_ = 293.1 J/g [68])_._ The crystallinity, *α*_c_, can be estimated by

(3) $$\alpha_{c}=\frac{\Delta h_{m}}{\Delta h_{\mathrm{PE}}\;\left(1-\alpha_{\mathrm{ACN}}\right)}$$

where Δ*h*_m_ is the measured specific melting enthalpy and α_ACN_ is the mass content of ACN monomers (0.34). The crystallinity after cooling in conventional DSC is 2.4%. After annealing for 600 min at −10 °C, it is *α*_c_ = 5.7%. The relatively low crystallinity is a consequence of the chemical confinement of the comonomers along the polymer chain [50].

The tetra-methylene or di-ethylene units in HNBR have the tendency to crystallize if the statistically generated sequences are long enough. The related homopolymer is polyethylene with the equilibrium melting temperature *T*_A_^0^ = 414.6 K and the equilibrium melting enthalpy Δ*h*_A_^0^ = 8.44 kJ/mol [68]. For the mole fraction of the tetra-methylene units, *X*_A_ = 0.646, the Flory model (Equation (1)) predicts an equilibrium melting temperature of *T*_eq_ = 79 °C. It should be noted that the Flory model is based on equilibrium thermodynamics. As a result of the metastable structure, polymer melting occurs significantly below the equilibrium melting temperature. The second monomer in HNBR is acrylonitrile. Homopolymers of acrylonitrile have a relatively low crystallinity (ca. 25 %) and a glass transition temperature of about 100 °C [69].

A crystallization of polybutadiene is only observed at high amounts of cis1.4 units (>98%). Due to the polymerization process of NBR, the cis1.4 content of the polybutadiene units is limited to a maximum of ca. 80%. Therefore, NBR is fully amorphous. The glass transition temperature and intensity of NBR with 34 wt % ACN is *T*_g_ = –22.5 °C and Δ*c_p_* = 0.5 J/(g K).

#### 3.1.2. The Rigid Amorphous Fraction in HNBR

According to Wunderlich et al. [6,7], semi-crystalline polymers can be separated in three fractions: the mobile amorphous fraction (MAF), the rigid amorphous fraction (RAF), and the crystalline fraction (CF). The RAF forms during the crystallization process due to the hindered mobility of chain segments, which are partially incorporated in the crystals. If *γ* and *δ* are the content of MAF and RAF, mass conversation yields

(4) $$1=\alpha_{c}+\delta +\gamma$$

The ratio between RAF and CF, *δ*/*α*_c_, depends on the crystalline structure [20,70]. During the diffusion-controlled crystallization of small crystalline structures, e.g., the mesophase crystallization in isotactic polypropylene (iPP) at low temperature [71], the ratio RAF/CF can be considered as constant. In this case, the formation of RAF during crystallization terminates the crystal growth. RAF acts as a self-assembled confinement [58].

To prevent the influence of structural relaxation on the endothermal melting peaks, HNBR was crystallized above the glass transition temperature at −10 °C. The sample was cooled with 20 K/min from 100 °C to the crystallization temperature and annealed for 0 min ≤ *t*_a_ ≤ 600 min. Subsequently, the sample was cooled with 20 K/min to −80 °C, equilibrated for 15 min, and then heated up to 100 °C with a heating rate of *β =* 10 K/min. The heating curves are displayed in Figure 2. The low melting temperature (close to the glass transition) indicates the formation of thermodynamically unstable, small crystallites.

**Figure 3:**
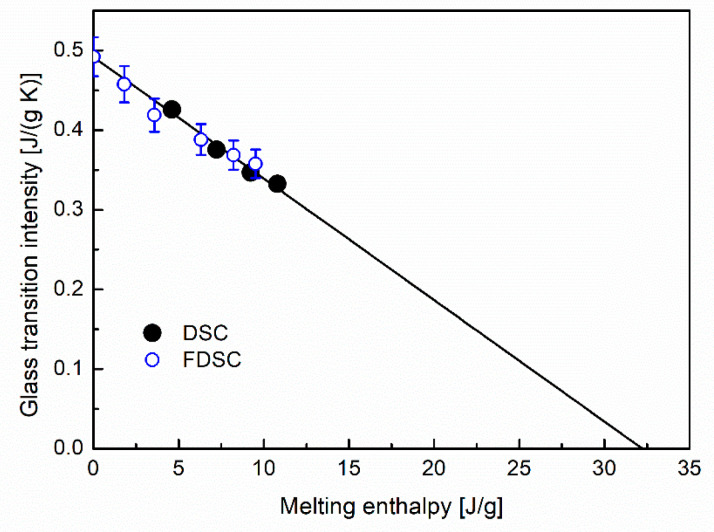
Glass transition intensity, Δ*c_p_*, versus specific melting enthalpy, Δ*h*_m_, for HNBR crystallized at −10 °C. The data are measured by DSC (solid dots) and FDSC (open dots).

The properties *γ* and *α*_c_ are determined from the intensity of the glass transition, Δ*c_p_*, and the melting enthalpy, Δ*h*_m_.. In HNBR, the glass transition and melting ranges overlap each other (Figure 2). This complicates the evaluation of Δ*c_p_* and Δ*h*_m_. Therefore, a new evaluation procedure was developed. This procedure is described in Appendix A. The data of Δ*c_p_* are plotted versus Δ*h*_m_ in Figure 3. This diagram shows a linear behavior

(5) $$\Delta c_{p}=\Delta c_{p1}-a\;\Delta h_{m}$$

with a negative slope *a* = 0.015 K^−1^ and an intercept of Δ*c_p_*_1_ = 0.49 J/(gK). The latter value is the glass transition intensity of a completely amorphous sample. Equation (5) can be derived from Equation (4) assuming that the ratio between RAF and CF is constant

(6) $$\delta =\sigma \cdot \alpha_{c}$$

*σ* is the contribution of the non-crystallizable rigid material in relation to the crystallinity. The chain segments in the RAF are characterized by a significantly hindered mobility. For this reason, these segments have no contribution to the glass transition. The amount of MAF, *γ*, is defined by

(7) $$\gamma =\frac{\Delta c_{p}}{\Delta c_{p1}}\;$$

Combining Equations (2), (4), (6), and (7) leads to the linear equation

(8) $$\Delta c_{p}=\Delta c_{p1}-\frac{\Delta c_{p1}\left(1+\sigma \right)}{\Delta h_{\mathrm{PE}}\left(1-\alpha_{\mathrm{ACN}}\right)}\;\Delta h_{m}.$$

A comparison of the slopes in Equations (5) and (8) yields a constant value *σ* = 4.9. This value is surprisingly high. For instance, in 1.4-cis-polybutadiene, *σ* is 0.9 [58]. The value of the mesophase of iPP is between 0.7 and 1.0 [71].

The segregation of ACN segments due to the crystallization of tetra-methylene segments could be a reason for the large amount of RAF. The remaining ACN-enriched regions have an increased glass transition temperature and are therefore part of the RAF.

From Equation (4) and (6), the crystallinity can be deduced

(9) $$\alpha_{c}=\frac{1-\gamma }{1+\sigma }\;$$

If no MAF exists (*γ* = 0), the maximum crystallinity is *α*_c_ = 16.9%. This is significantly higher than the measured value. Since there are always sections with short sequences of alternating ACN and tetra-methylene segments, which cannot crystallize, these sections contribute to the MAF. The low melting temperature and the relatively low degree of crystallinity is a consequence of a kinetically hindered crystallization due to the simultaneous formation of large amounts of RAF.

### 3.2. FDSC Measurements and Data Evaluation

#### 3.2.1. The Temperature Program of the FDSC Measurements

We wanted to measure the sequence of structural relaxation and crystallization during isothermal annealing around the glass transition. However, the heat release of these processes is so low that it cannot be measured directly. We interrupted the annealing process by fast cooling and measured the enthalpy change during annealing by analyzing related peaks during a subsequent heating segment. The origin of the peaks could be enthalpy relaxation or melting.

**Figure 4:**
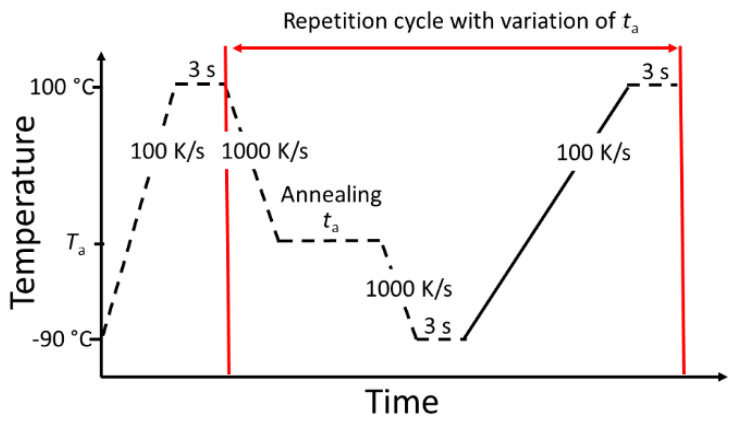
Typical temperature program for the Fast Differential Scanning Calorimetry (FDSC) measurements.

A sketch of the temperature program for FDSC measurements is shown in Figure 4. Initially, the sample is heated to 100 °C and stabilized for 3 s. To avoid crystallization, the sample is afterwards cooled with 1000 K/s to the annealing temperature *T*_a_. At this temperature, the sample is isothermally stored for a certain annealing time *t*_a_. To interrupt the relaxation and crystallization, the sample is subsequently cooled with 1000 K/s to −90 °C. After equilibration, the sample is heated with 100 K/s to 100 °C. This heating segment is analyzed to determine the enthalpy change during the annealing step. The sequence between the red lines in Figure 4 is repeated for various annealing times (0 s ≤ *t*_a_ ≤ 5000 s) and temperatures (−50 °C ≤ *T*_a_ ≤ 10 °C).

#### 3.2.2. Enthalpy Determination

**Figure 5:**
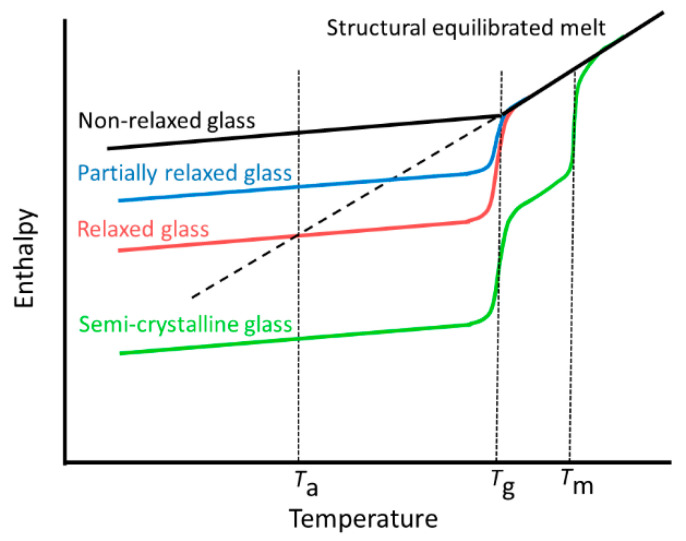
Sketch of the enthalpy–temperature behavior of differently annealed glasses at the annealing temperature *T*_a_.

The change of enthalpy during annealing below the glass transition is schematically shown in Figure 5. During the cooling of a structural equilibrated super cooled melt, the glass transition occurs in the temperature range around *T*_g_. The enthalpy in the glassy state is larger than the enthalpy of the supercooled melt at the same temperature. This enthalpy difference increases with decreasing temperature. During annealing at *T*_a_ < *T*_g_, structural relaxation occurs, and the enthalpy of the glass decreases. This enthalpy change causes the enthalpy relaxation peak during heating. The enthalpy of a completely relaxed glass corresponds to that of the structural equilibrated supercooled melt at *T*_a_. This is the maximum possible enthalpy change due to the structural relaxation of a glass. A larger enthalpy change is a clear indication for crystallization during annealing.

The enthalpy change Δ*H*_a_ is the difference of the enthalpy between the annealed and the non-annealed sample below *T*_a_. In terms of heat flow, this is:

(10) $$\Delta H_{a}\left(T_{a},t_{a}\right)=\frac{1}{\beta }\;\overset{T_{2}>T_{g}\;\mathrm{or}\;T_{m}}{\underset{T_{1}<T_{a}}{\int }}\left(\Phi \left(T,t_{a}\right)-\Phi \left(T,t_{a}=0\right)\right)\;dT\;$$

**Figure 6:**
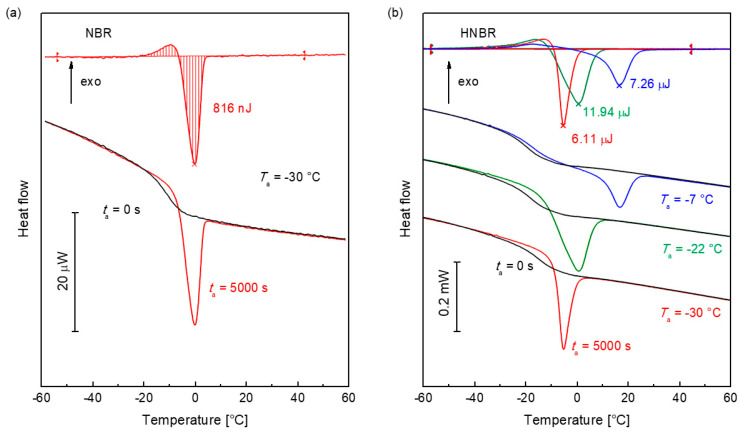
Determination procedure of the enthalpy change during annealing. (**a**) Measurement of the enthalpy relaxation of amorphous NBR. The lower curves are measured after annealing at −30 °C for 0 s (black) and 10,000 s (red). The upper curve is the differences of both curves. The enthalpy was determined by integration. (**b**) Evaluation of the curves of semicrystalline HNBR after annealing at –30 °C, –22 °C, and −7 °C. The measurement curves after 0 s and 5000 s are shown below. The black curves are measured without annealing. The upper curves show the differences, which were used for the evaluation of the enthalpy by integration.

This is illustrated in Figure 6. As an example, Figure 6a displays FDSC curves of the non-relaxed (black) and the 10,000 s at −30 °C annealed (red) NBR sample. The upper curve is the difference of both. An integration (see Equation 10) of the difference curve yields the enthalpy of relaxation. Figure 6b shows curves for HNBR, which was annealed at −30 °C, −22 °C, and −7 °C.

The maximum enthalpy of the structural relaxation, Δ*H*_max_, was determined from cooling curves,

(11) $$\Delta H_{\max}\left(T_{a}\right)=\frac{1}{\beta }\;\overset{T_{2}>T_{g}}{\underset{T_{a}}{\int }}\left(\Phi \left(T\right)-\Phi_{\mathrm{liquid}}\left(T\right)\right)\;dT\;$$

where Φ_liquid_ is the extrapolated heat flow above the glass transition, and Φ is the measured heat flow. Due to the absence of an enthalpy overshoot, cooling curves are preferred for the determination of Δ*H*_max_.

**Figure 7:**
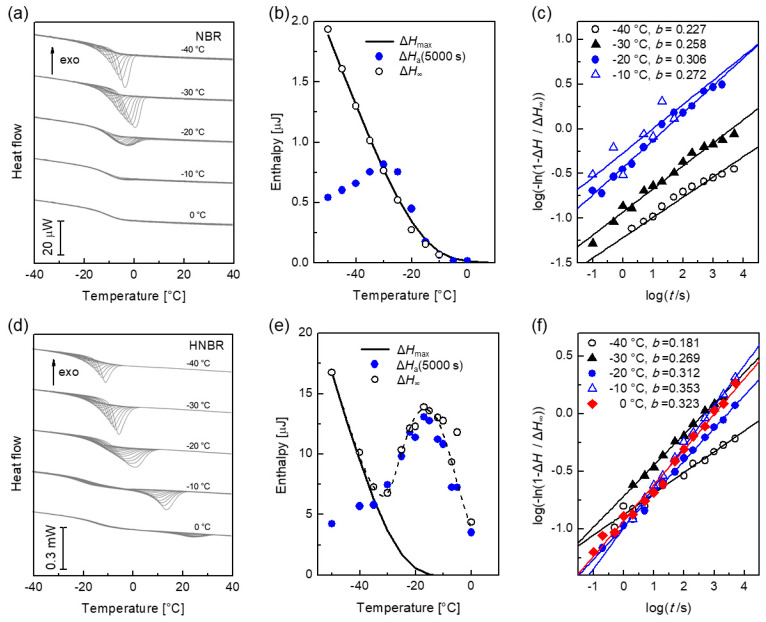
Measured annealing behavior of NBR (above) and HNBR (below) at selected annealing temperatures: (**a**) measurement curves of NBR. (**b**) Enthalpy as a function of temperature: solid circles are the enthalpy after annealing for 5000 s, the open circles are the maximum enthalpy for the curve fit with Equation (12). The curve is the maximum enthalpy determined from the FDSC cooling curve with Equation (11). (**c**) Representation of the enthalpy change during annealing in the linearized diagram (Equation (13)). (**d**) Measurement curves of HNBR. (**e**) Enthalpy versus temperature. (**f**) Linear representation of the enthalpy change during annealing.

The maximum relaxation enthalpy, Δ*H*_max_(*T*_a_) for NBR at −30 °C of 0.77 μJ, agrees with the measured value in Figure 6a of 0.816 μJ (see also Figure 7b). The glass is completely relaxed. For HNBR, the values of Δ*H*_max_ and Δ*H*_a_ for *t*_a_ = 5000 s are listed in Table 1. At −30 °C, HNBR is completely relaxed at *t*_a_ =5000 s (Δ*H*_a_ ≈ Δ*H*_max_). After annealing at *T*_a_ = –22 °C, Δ*H*_max_ is less than Δ*H*_a_ (5000 s). The measured peak is a superposition of enthalpy relaxation and melting. Above the glass transition temperature at –7 °C, HNBR only crystallizes.

#### 3.2.3. Kinetic Evaluation

The enthalpy change during isothermal annealing is usually modeled by a stretched exponential function

(12) $$\Delta H_{a}\left(t_{a}\right)=\Delta H_{\infty }\left(1-\exp\left(-{\left(\frac{t_{a}}{\tau }\right)}^{b}\right)\right)$$

where Δ*H*_∞_ is the maximum enthalpy change of the occurring process, *τ* is the characteristic time, and *b* is the stretched exponent. Equation (12) describes both relaxation and crystallization. For 0 < *b* ≤ 1, this equation is the Kohlrausch function [72,73], which is used for relaxation processes. If 1 ≤ *b*, the equation is the Kolmogorov–Johnson–Mehl–Avrami (KJMA) equation, which describes the kinetics of crystallization [74,75,76]. The rate constant in the KJMA equation is *k* = *τ*^-*b*^.

A linearization of a stretched exponential (Equation (12)) yields

(13) $$\log\left(-\ln\left(1-\frac{\Delta H_{a}\left(t_{a}\right)}{\Delta H_{\infty }}\right)\right)=-b\log\tau +b\log t_{a}$$

One might expect that three processes could occur during annealing. The first is the structural relaxation [77,78], the second is the primary crystallization, and the third is the secondary crystallization [79]. For a couple of homopolymers, it was shown that nucleation and crystallization follow the structural relaxation during annealing below and in the glass transition region [42]. In this paper, we want to clarify whether this finding can be generalized for polymers with chemical confinements due to the molecular structure. In this case, the annealing kinetics should be described by the sum of three stretched exponential functions.

DSC curves of an amorphous NBR are shown in Figure 7a. The enthalpy change Δ*H*_a_ (5000 s) is shown in Figure 7b (filled blue dots) as a function of *T*_a_. For annealing below −30 °C, the annealing time of 5000 s is not sufficient to reach the structural equilibrated liquid with Δ*H*_a_ = Δ*H*_max_ (solid curve in Figure 7b). At *T*_a_ > −30 °C, Δ*H*_a_ (5000 s) agrees with Δ*H*_max_. In the glass transition region (0 °C ≥ *T*_a_ ≥ -10 °C), the relaxation becomes fast (about 20 s), and the enthalpy change is small. Above the glass transition, no relaxation occurs. The parameters of the stretched exponential function (Equation (12)) Δ*H*_∞,_ *τ* and *b* are determined by a last-square fit. Δ*H*_∞_ for different temperatures is included in Figure 7b and agrees with Δ*H*_max_ (open dots). The linearized diagram according to Equation (13) is plotted in Figure 7c. A linear function in this diagram is a good indication that the structural relaxation follows the stretched exponential behavior. The slope corresponds to 0.23 ≤ *b* ≤ 0.31. The Kohlrausch exponent, *b*, increases slightly with increased annealing temperature.

Figure 7d shows DSC curves of HNBR for selected temperatures between −40 and 0 °C. An exothermal peak occurs also after annealing above the glass transition range (*T*_a_ > −15 °C). This indicates crystallization during annealing. The solid curve in Figure 7e is the maximum enthalpy due to structural relaxation, Δ*H*_max_ (Equation (11)). The measured enthalpy after annealing for 5000 s is displayed with solid circles. The open circles represent Δ*H*_∞_. Below −30 °C, Δ*H*_a_ (5000 s) is smaller than Δ*H*_max_ and Δ*H*_∞_ matches with Δ*H*_max_. In this temperature range, the measurement behavior can be described completely by structural relaxation. Above −30 °C, Δ*H*_a_ (5000 s) is slightly lower than Δ*H*_∞_ but significantly larger than Δ*H*_max_.

The linearized diagram in Figure 7f is sensitive to the variation of the Kohlrausch exponent. In the case of successive relaxation and crystallization, a change of the slope is expected. If nucleation occurs after the structural relaxation is completed (this is the case described in reference [42]), the measured curve should behave in a non-linear manner, since the parameter *b* tends to change from a typical Kohlrausch exponent to zero. If crystallization follows the structural relaxation directly, the slope will increase according to the change of *b* to a value of a typical Avrami exponent.

However, the curves in Figure 7f always show a constant slope, which is slightly increased with increasing *T*_a_. This behavior is also observed in temperature ranges where no structural relaxation (*T*_a_ ≥ −15 °C) occurs. The slope is always below 0.4, which is not expected for crystallization.

**Figure 8:**
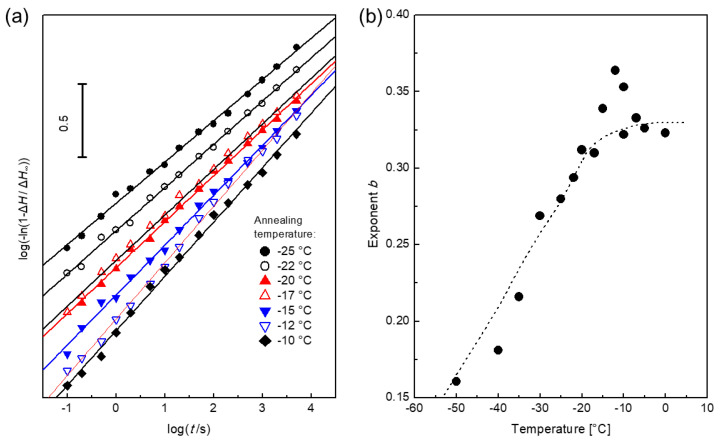
Data of H34. (**a**) Linearized diagram of the stretched exponential function in the temperature range between −25 and −10 °C. For a clearer visibility, the graphs are shifted in the direction of the ordinate with increasing temperature by an increment of −0.1. (**b**) The stretching exponent *b* versus the annealing temperature. The dashed line is a guide for the eyes.

The behavior during annealing at temperatures in the glass transition range (between −25 and −10 °C) is shown in Figure 8a. The displayed curve are consecutively shifted downwards by −0.1 on the ordinate with increasing *T*_a_ to achieve a more concise visualization. The annealing kinetics show no transition from structural relaxation to crystallization. In Figure 8b, the exponent *b* is plotted versus the annealing temperature. The exponent increases with increasing temperature. Above the glass transition range (*T*_a_ > −15 °C), the exponent seems to reach a constant value of approximately 0.35.

At *T*_a_ ≥ −15 °C, Figure 7e indicates a steep decrease of the crystallinity and an increased crystallization temperature. The reason could be an increased critical crystalline size with increasing temperature. The reduced number of sufficiently long tetra-methylene sequences reduces the maximum crystallinity at higher temperature.

#### 3.2.4. The Avrami Exponent and the Model for Relaxation and Overall Crystallization

HNBR crystallizes during annealing between −30 and 0 °C (Figure 7e and Figure 8b). The kinetics follows the KJMA equation with an Avrami exponent 0.25 ≤ *b* ≤ 0.40. Such small values are usually not observed. Before we discuss this finding, we briefly review the interpretation of the Avrami exponent *b*. Evens discussed the case of a constant growth rate (*R* ∝ *t*, *R* is the particle size) [80]. As a result, the exponent can be expressed by an integer between 1 and 4 depending on the growth dimension and the mechanism of nucleation. Turnbull showed that in this case, the growth is surface controlled [81]. If concentration gradients occur in the surrounding of the formed particles, the growth rate is reduced with increasing time according to *R* ∝ (*D t*)^1/2^ (where *D* is the diffusion coefficient in the liquid matrix) [81,82]. Thermal diffusion behaves accordingly. In the case of local overheating due to fast crystallization with a high heat release and/or low thermal diffusivity *κ*, the growth rate *R* is proportional to (*κ t*)^1/2^ [83]. This reduces *b* by a factor of 1/2. We summarize this in a general expression of the Avrami exponent

(14) $$b=\frac{n_{g}}{n_{d}}+n_{n}\;$$

where *n*_g_ is the dimension of growth (1, 2 or 3), *n*_n_ describes the kind of nucleation (1 for instantaneous or heterogeneous and 2 for spontaneous or homogeneous nucleation), and *n*_d_ characterizes the influence of diffusion on the growth rate. For a constant growth rate, *n*_d_ is 1. In the case of diffusion control, *n*_d_ is 2.

According to Equation (14), the smallest possible Avrami exponents is *b* = 0.5 for heterogeneous nucleated, diffusion controlled, one-dimensional crystallization. Avrami exponents in the order of 0.5 are measured in block copolymers as consequents of confinements due to phase segregation [84,85,86]. Such values are interpreted as the result of processes between sporadic and instantaneous nucleation [84].

Avrami exponents in the order of 0.3 are measured for thermotropic copolyesters [87,88]. These materials consist of rigid macromolecules without chain folding and anisotropic molecular motion in the supercooled melt. The small exponents are interpreted as the consequence of a restricted mobility due to previously formed crystals [87] or of a crystallization with a limited size of the crystallites [88].

In the system that we characterized, the macromolecules are flexible. Due to the limited length of the tetra-methylene sequences, the formation of folding crystals is unlikely. The statistic distribution of the comonomers along the polymer chain creates chemical confinements. As a consequence, the formation of a large amount of RAF occurs with crystallization.

The curves in Figure 8a show no difference between structural relaxation and crystallization. This cannot be explained with the common interpretation of the Avrami exponents. Our preferred model assumes an identical molecular rearrangement process for the structural relaxation and the crystallization. A crystalline structure with a critical size and the surrounded RAF is formed randomly. On the one hand, the RAF stabilizes the crystalline structure; on the other hand, it limits any further growth. The reduced molecular mobility and the reduced concentration of crystallizable sequences in the amorphous fraction decrease the possibility of the formation of further crystalline structures. For such a process, there is no difference among structural relaxation, nucleation, and crystallization, since the origin and kinetics of all processes are based on the same restricted molecular rearrangements. This model also explains the relatively low crystallinity and melting temperature.

#### 3.2.5. The Crystallization Half Time

It is common to characterize crystallization processes with the half time *t*_1/2_,

(15) $$t_{1/2}=\tau \;\ln{\left(2\right)}^{1/b}$$

**Figure 9:**
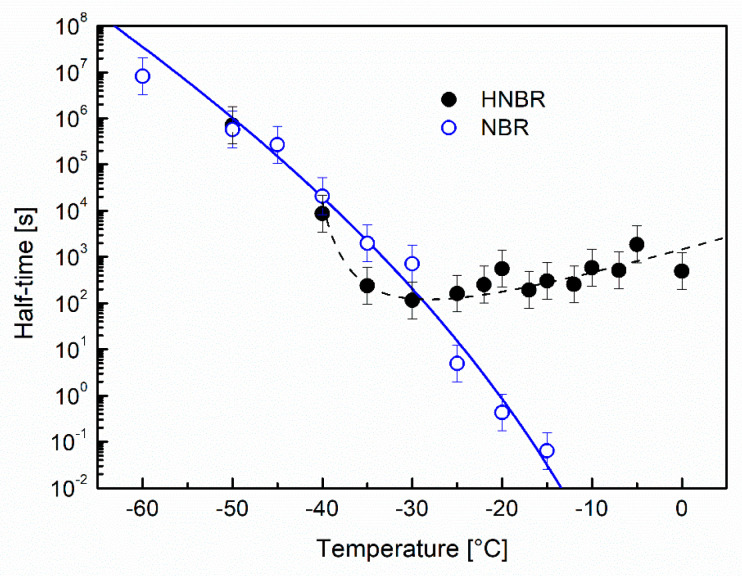
Half time of the annealing process as a function of the annealing temperature for NBR and HNBR. The curves just serve as an indication.

Figure 9 shows *t*_1/2_ as a function of the annealing temperature for NBR and HNBR.

NBR shows structural relaxation during annealing. The half time is a measure of the average relaxation time. The temperature behavior of *t*_1/2_ in Figure 9 is characterized by different curvature compared to the Vogel–Fulcher–Tammann behavior. This deviation is caused by a deviation from the structural equilibrium in the vitrified state [31,89].

During annealing below −35 °C, HNBR does not crystallize and behaves similar to NBR. For *T*_a_ ≥ –35 °C, the half time remains in the range between 100 and 1000 s, which slightly increases with temperature. This can be interpreted as the vitrification of RAF during crystallization. After the RAF is formed, it controls the molecular transport of crystallizable molecular segments to the growth face.

#### 3.2.6. Determination of RAF from FDSC Measurement

In contrast to conventional DSC, HNBR can be easily kept in an amorphous state by FDSC. In this section, we compare the RAF content and the kinetics of crystallization measured with both techniques. The FDSC measurement curves from Figure 7d for *T*_a_ = −10 °C were utilized for further evaluations due to the relatively large melting enthalpy and the fact that practically no enthalpy relaxation occurs.

The overlap of the glass transition and the melting in the FDSC curves does not allow the direct evaluation of Δ*c_p_* and Δ*h*_m_. The utilized procedure of evaluation is described in Appendix A. The results are plotted as open circles in Figure 3. These results are in good agreement with the DSC data. Both techniques yield the identical ratio between CF and RAF.

A difference between both techniques becomes evident if the crystallization rates are compared. The annealing time to reach an enthalpy of 10 J/g with the DSC measurements (ca. 300 min) is about four times longer than for the FDSC measurement (ca. 80 min). This discrepancy can be explained with initial structural differences before annealing. The relatively fast crystallization in the FDSC starts from a completely amorphous material. The initial DSC sample is partially crystalline due to the low cooling rate. The existence of RAF hinders the rearrangements on the growth front and delays further crystallization [90].

## 4. Conclusions

The influence of confinements in HNBR on the mechanism of crystallization was investigated. The confinements in HNBR are caused by the chemical structure due to the statistical arrangement of the comonomers in the polymer chain. The comparison of the intensity of the glass transition, Δ*c_p_*, and the melting enthalpy of semicrystalline HNBR shows that a rigid amorphous fraction (RAF) with reduced molecular mobility is formed during crystallization. The ratio between RAF and the crystalline fraction (CF) is significantly higher than for other polymeric nanophases (RAF/CF ≈ 5). This indicates that each crystallite is covered by an amorphous fraction with reduced mobility. In homopolymers, the mobility of chain segments in the vicinity of the crystals is hindered due to the incorporation of segments in the crystals. In random copolymers such as HNBR, the crystallization-induced segregation of ACN sequences yields increased RAF. The ACN-enriched regions have an increased glass transition temperature and therefore contribute to the RAF.

The high fraction of RAF indicates small crystallites. We assume that fringed micelles or crystalline nanoclusters are formed. The formation of small crystallites and a large amount of non-crystallizable material is also supported by the low melting temperature and enthalpy.

The measured melting enthalpy decreases significantly after annealing at *T*_a_ > −15 °C. At higher temperature, the critical size of the formed crystals increases. Consequently, the possibility of crystal formation due to a smaller number of sufficient long tetra-methylene sequences decreases. The large amount of RAF formed during crystallization stabilizes small crystallites and hinders further growth due to the reduced diffusion.

During annealing in the glass transition range, many homopolymers show a sequence of structural relaxation, nucleation, and crystallization [43]. Such a behavior was not measured in HNBR. In the glass transition, range structural relaxation and crystallization follow the same kinetics. Thus, a difference of both processes could not be found, and it seems that both processes occur simultaneously. The Avrami exponent (for crystallization) and the Kohlrausch exponent (for relaxation) are identical. The measured exponents are between 0.3 and 0.4. This means that in HNBR, only one single stretched exponential describes relaxation and crystallization. It can be concluded that the same molecular rearrangements control both the structural relaxation and the crystallization.

Below the glass transition (*T*_a_ < −40 °C), the HNBR behaves similar to the amorphous NBR: only structural relaxation occurs during annealing up to 5000 s. In the glass transition range of HNBR, relaxation and crystallization take place. The characteristic time of the overall process stabilizes at about 100 s and is nearly temperature independent. This could be interpreted as the vitrification of RAF.

## Figures and Tables

**Table 1:** Maximum annealing enthalpy Δ*H*_max_ (Equation (11)) and the measured enthalpy of annealing after Δ*H*_a_ (5000 s) (Figure 6b) for selected annealing temperatures of HNBR.

*T*_a_ (°C)	Δ*H*_max_ (μJ)	Δ*H*_a_ (5000 s) (μJ)
−30	6.66	6.11
−22	0.95	11.94
−7	0.00	7.26

**Figure A1:**
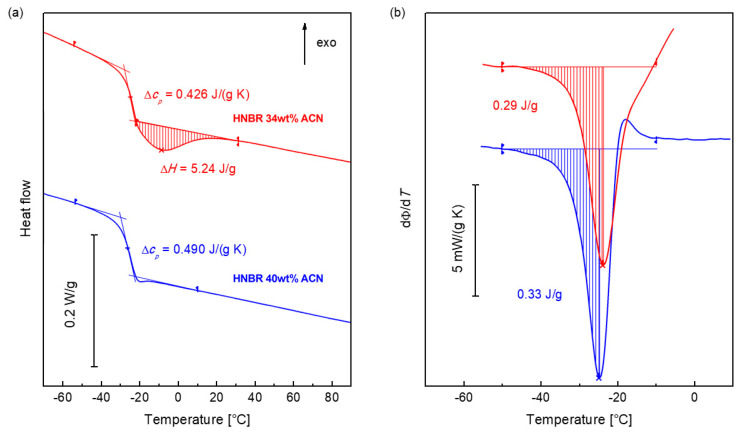
(**a**) DSC heating curves of HNBR with 34 wt % and 40 wt % ACN. (**b**) The first derivatives of the DSC curves in the glass transition range.

**Figure A2:**
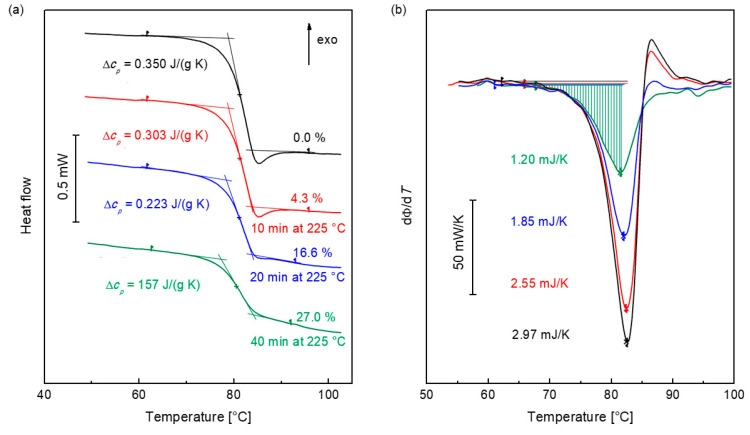
Curves of differently crystallized poly(ethylene terephthalate) (PET). (**a**) DSC curves in the glass transition range including glass transition evaluation. (**b**) Derivative of the curves from the left diagram and integration of the left side of the peak to determine Δ*c*_1/2_ according to Equation (A1).

## Appendix A

### Appendix A.1. General Comment about the Evaluation of the Glass Transition by Conventional DSC

For the determination of the glass transition temperature, *T*_g_, and the intensity of the glass transition, Δ*c_p_*, the extrapolated tangents of the heat flow curve below Φ_glass_(*T*) and above the glass transition range Φ_liquid_(*T*) are needed. The glass transition temperature, *T*_g_, is the temperature of the intersection point of the DSC curve with the bisecting line of the two tangents (Φ_glass_(*T*) and Φ_liquid_(*T*)). The intensity of the glass transition, Δ*c_p_*, is usually determined form the vertical distance between the two extrapolated tangents at *T*_g_:

(A1) $$\Delta c_{p}=\frac{\Phi_{\mathrm{liquid}}\left(T_{g}\right)-\Phi_{\mathrm{glass}}\left(T_{g}\right)}{\;m\;\beta \;}$$

where *m* and *β* are the sample mass and the scanning rate.

In our opinion, it is important to mention the method of the determination of Δ*c_p_*, because several methods are specified in standards that differ in content from Equation (A1). These methods describe the shape of the glass transition, but they cannot be used for a quantitative analysis.

### Appendix A.2. Determination of Δc_p_ and Δh_m_ for HNBR

The DSC curve of HNBR in Figure 2 show an overlap between the glass transition and the melting peak. This makes it difficult to find the best baseline for the peak integration and the correct high temperature tangent, Φ_glass_(*T*), for the evaluation of the glass transition.

HNBR with 40 wt % ACN (Zetpol 2001 from Zeon Corp., Tokyo, Japan) does not crystallize during cooling at 20 K/min. This material was used to determine the glass transition intensity Δ*c_p_*_,0_ = 0.49 J/(g K) of completely amorphous HNBR. The DSC heating curves of both types of HNBR are shown in Figure A1 (left). In DSC heating curves, the glass transition, measured at rates that are not much higher than the rates of the subsequent cooling process, shows no considerable enthalpy relaxation peak. The temperature of the inflection point of the measured glass transition step is then nearly *T*_g_. We assume that CF and RAF reduce the glass transition intensity in such a way that the ratio of the step height before and after the inflection point is not changed. This assumption is successfully tested in Appendix A.3.

In HNBR (with 34 wt % ACN), the melting peak appears at the high temperature side of the glass transition and does not influence the shape of the glass transition below the inflection point (Figure A1a). The height of the heat capacity step between a temperature in the glassy state and the inflection temperature, Δ*c*_1/2_, can be determined by integration of the first derivative of the DSC curve using a horizontal baseline:

(A2) $$\Delta c_{1/2}\left(t_{a}\right)=\frac{1}{\beta }\overset{T_{2}}{\underset{T_{1}}{\int }}\left(\frac{d\Phi (t_{a})}{dT}\right)dT$$

where *T*_1_ and *T*_2_ are a temperature below the glass transition range and the temperature of the extremum of the derivative of the heat flow curve in the glass transition range. For non-crystalline and semi-crystalline HNBR, this integration is shown in Figure A1b. The glass transition intensity is

(A3) $$\Delta c_{p}\left(t_{a}\right)=\frac{\Delta c_{1/2}\left(t_{a}\right)}{\Delta c_{1/2,0}}\;\Delta c_{p,0}$$

where Δ*c*_1/2,0_ is the value of Δ*c*_1/2_ for non-crystallized material.

The evaluation of the glass transition and the melting peak is shown in the example of the semicrystalline HNBR (with 34 % ACN) in Figure A1b. From Equation (A3) follows Δ*c_p_* = 0.43 J/(g K). The high temperature limit of the tangent Φ_liquid_(*T*) is selected shortly above the end of the melting peak. The slope of this tangent is varied in such a way that the resulting glass transition intensity agrees with the calculated Δ*c_p_*. This tangent is also taken as the baseline for the enthalpy determination. The lower integration limit is selected as the intersection of this tangent with the heat flow curve. In the example in Figure A1, the melting enthalpy is Δ*h*_m_ = 5.24 J/g.

### Appendix A.3. Validation of the evaluation method using poly(ethylene terephthalate) as an example

Poly(ethylene terephthalate) (PET) is a relatively slowly crystallizing polymer with a glass transition temperature at about 70 °C. After crystallization at 225 °C and subsequent rapid cooling, cold crystallization and melting occur well separated from the glass transition. Consequently, PET can be used as a material to investigate the evaluation method mentioned above.

The PET is taken from the Tutorial Kid for Thermal Analysis (from Mettler-Toledo, Nänikon, Switzerland). The sample was crystallized at 225 °C for 0 min, 10 min, 20 min, and 40 min. After crystallization, the sample was taken out of the furnace and placed on an Al-plate at RT. In the subsequent heating run, the glass transition, crystallization, and melting were measured. The crystallinity was determined by integration of the crystallization and the melting peak with a single baseline [91]. The glass transition evaluation is displayed in Figure A2a.

The determination of Δ*c*_1/2_ from the derivative of the DSC curve is shown in Figure A2b. From Δ*c*_1/2_, the intensity of the glass transition, Δ*c_p_*, is determined with Equation (A3) and listed in Table A1. The measured and calculated values of Δ*c_p_* agree well at least up to a crystallinity of about 15%. This is sufficient for the analysis of HNBR and indicates the applicability of the used procedure.

**Table A1:** Glass transition intensity of differently crystallized PET at 225 °C. The measured Δ*c_p_* is determined from Figure A2a. Δ*c*_1/2_ is determined by integration of the low-temperature side of the first derivative in Figure A2b. The calculated data are determined by Equation (A3).

Crystallinity [%]	Δ*c_p_* [J/(g K)] measured	Δ*c*_1/2_ [mJ/K]	Δ*c_p_* [J/(g K)] Calculated
0.0	0.350	2.97	-
4.3	0.303	2.55	0.300
16.5	0.223	1.85	0.218
27.0	0.157	1.20	0.141
